# Immune Effects of γδ T Cells in Colorectal Cancer: A Review

**DOI:** 10.3389/fimmu.2020.01600

**Published:** 2020-09-09

**Authors:** Rulan Ma, Dawei Yuan, Yizhan Guo, Rong Yan, Kang Li

**Affiliations:** ^1^Department of Surgical Oncology, The First Affiliated Hospital of Xi’an Jiaotong University, Xi’an, China; ^2^Department of Surgery, University of Virginia, Charlottesville, VA, United States

**Keywords:** γδ T cells, colorectal cancer, antitumor effects, protumor effects, immunotherapy

## Abstract

Gamma delta (γδ) T cells can effectively recognize and kill colorectal cancer (CRC) cells, thereby suppressing tumor progression *via* multiple mechanisms. They also have abilities to exert a protumor effect *via* secreting interleukin-17 (IL-17). γδ T cells have been selected as potential immunocytes for antitumor treatment because of their significant cytotoxic activity. Immunotherapy is another potential anti-CRC strategy after an operation, chemotherapy, and radiotherapy. γδ T cell-based immunotherapy for CRC shows fewer side effects and better toleration. This review will outline the immune functions and the mechanisms of γδ T cells in the growth and progression of CRC in recent years, and summarize the immunotherapies based on γδ T cells, thus providing a direction for future γδ T cells in CRC research.

## Introduction

Colorectal cancer (CRC) is one of the most common gastrointestinal malignant tumors in the world, with a high therapeutic need. Globally, CRC is the third most frequently diagnosed cancer and the second leading cause of death from tumor diseases (1). The incidence and mortality of CRC are rising rapidly in many low- and middle-income countries (2). In some high-income countries, although the overall incidence and mortality of CRC has declined or stabilized (the decline of CRC incidence and mortality in the older age group is partly due to the implementation of CRC screening), the CRC incidence and mortality in individuals under 50 has increased significantly (3, 4). Presently, surgery remains the primary strategy to treat advanced CRC. Even though advances in screening, diagnosis, and treatment have improved the prognosis of CRC in some countries, there are still many people (about 25%) that are diagnosed with advanced CRC, among which almost 50% develop metastases (5, 6). Only a few patients diagnosed with advanced CRC can receive radical surgeries (7), and resection of resectable liver and lung metastases offers 20–45% 5-year survival rates in carefully selected individuals (6). The overall efficacy of surgery and adjuvant therapy in these individuals is not satisfactory. It has been shown that most patients (71.2%) with advanced CRC develop recurrence within the first 2 years after surgery and the 5-year overall survival rate of these patients was 34.7% (8). It is becoming more evident that the components of the tumor microenvironment (TME) play critical roles in the occurrence, development, and prognosis of CRC (9, 10). For example, NK cells (one of the immunocyte subsets in TME) that can combine HLA class I molecules are able to recognize and eliminate CRC cells with aberrant HLA class I expression (11). Cytokines, like IL-17, have abilities to perpetuate CRC progression *via* promoting angiogenesis and the production of myeloid-derived suppressor cells (MDSCs) (12). Moreover, a meta-analysis showed a statistically significant inverse relationship between intratumoral vessel density (a surrogate marker of tumoral angiogenesis) or vascular endothelial growth factor (one of the proangiogenic factors in TME) expression and overall survival in CRC (13). Therefore, it is necessary to discover a novel reasonable treatment strategy based on the understanding of the TME.

Gamma delta (γδ) T cells are one small population of the TME and a small subset of peripheral blood T lymphocytes, which express heterodimeric receptor composed of γ and δ chains on the cell surface. Most γδ T cells are CD4^–^ or CD8^–^ T cells, while αβ T cells express CD4^+^ or CD8^+^ molecules. γδ T cells are mainly distributed in subcutaneous tissue, the mucosa of the intestinal tract, respiratory tract, and urogenital tract. They are involved in the composition of intestinal intraepithelial lymphocytes. Since γδ T cells recognize infected and transformed cells rapidly, they are recognized as the first line of defense against infections and malignant tumors (14). γδ T cells have pleiotropic biological effects, including exerting cytotoxicity to kill tumor cells, involved in immune regulation, presenting antigen, inducing dendritic cells (DCs) maturation, and so on (15, 16). Moreover, γδ T cells are capable of infiltrating into various tumor tissues, like rectal cancer, breast cancer, or pancreatic cancer (17–19). In addition, they have abilities to recognize different tumor cells in a major histocompatibility complex (MHC)-unrestricted manner and lyse cancer cells by producing chemokines and cytokines, or by direct contact with cancer cells through the death receptor signal (19, 20), which suggests that γδ T cells may have a potential role in antitumor immunotherapy. However, accumulating evidence suggests that γδ T cells also play a protumor role mainly by expressing interleukin-17 (IL-17) in several cancers (17, 21–26).

Reviews published in the past few years mainly discuss the functions of γδ T cells and their immunotherapeutic potential against cancer in general. However, only a few foci were placed on the immune effects and immune treatment strategies of γδ T cells against CRC. In this review, we will summarize recent advances in the role of γδ T cells in CRC immunity, as well as γδ T cells-based immunotherapies against CRC.

## γδ T Cells

**FIGURE 1:**
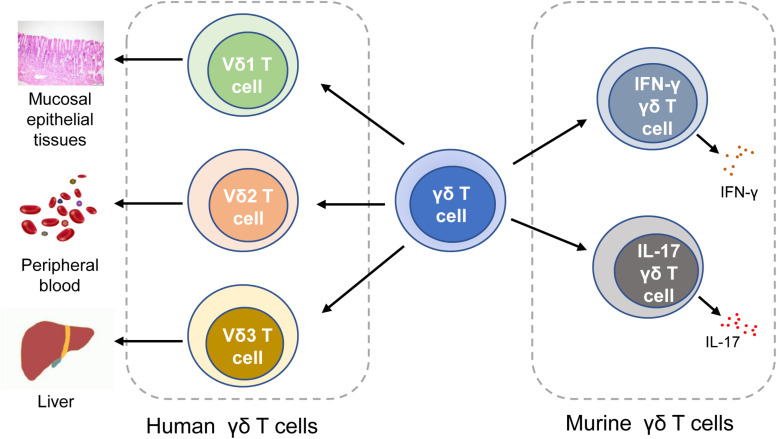
Classification of γδ T cells. In human, γδ T cells can be classified into Vδ1 T cells, Vδ1 T cells, and Vδ3 T cells. In mice, γδ T cells can be categorized into IFN-γγδ T cells and IL-17 γδ T cells. IFN-γ, interferon-γ.

Human γδ T cells are classified into three subtypes based on the expression of δ chains: (1) Vδ1 T cells are enriched in the thymus and mucosal epithelial tissues. They produce a variety of cytokines like TNF-α and IFN-γ, and lyse infected or transformed target cells by cytotoxicity (20, 27). (2) Vδ2 T cells, which exert cytotoxicity in tumor immunoregulation and viral infection, are mainly distributed in peripheral blood (28). T cell receptors (TCRs) expressed on the surface of Vδ2 T cells are able to recognize phosphoantigens produced by malignant cells, thereby activating Vδ2 T cells to release perforins, granzymes, and IFN-γ (20, 29). MDSCs may inhibit the production of IFN-γ and degranulation in phosphoantigens-activated Vδ2T cells (30). (3) Vδ3 T cells are mainly distributed in the liver tissue and rarely in the peripheral blood, which can directly kill the target cells and secrete cytokines. In mice, γδ T cells are categorized into two subtypes based on what cytokines they release. Murine IFN-γγδ T cells express the CD27 molecule (which is a member of the TNF receptor family and can bind to CD70), whereas IL-17 γδ T cells cannot display CD27 (31). In addition to mice, IL-17 γδ T cells are also found in humans (32–34). Although the production of IL-17 by human γδ T cells is rare, it is involved in the initiation and progression of CRC by producing growth factors (12, 32). Pin Wu et al. previously reported that tumor-infiltrating IL-17 γδ T cells might be a key player in human CRC progression and metastasis (32). It is also reported that IL-17 perpetuate CRC progression *via* promoting angiogenesis and the IL-17/IL-23 pathway plays a critical role in pathogenesis of CRC (IL-23 is a key modulator of IL-17 γδ T cells responses) (12) (Figure 1).

### Vδ2 T Cells

Vδ2 T cells are the most abundant subset of γδ T cells, accounting for 50 to 90% of the total number of γδ T cells. Among Vδ2 T cells, Vγ9Vδ2 T cells that co-express Vδ2 and Vγ9 chains are the most abundant subtype. Vγ9Vδ2 T cells elicit robust inhibitory effects on the tumorigenesis and growth of different tumors (35–37). Murielle Corvaisier et al. (38) found that colon tumor is frequently infiltrated by Vγ9Vδ2 T lymphocytes. Moreover, a recent analysis of expression signatures from 39 different malignancies, including CRC, revealed that tumor-infiltrating γδ T cell is one of the most significantly favorable cancer-wide prognostic immunocytes (39). Later, Liang Rong et al. (17) used the Application of Fluorescent-activated Cell Sorting (FASC) to analyze the percentage of γδ T cells (including Vδ1 T cells and Vδ2 T cells) in tumor tissues, para-carcinoma tissues and peripheral blood from 20 rectal cancer patients. The results showed that although the percentage of Vδ2 T cells in tumor tissues was negatively correlated with the T staging, the tumor-infiltrating Vδ2 T cells showed robust cytolytic activity. Recently, an assessment of tumor-infiltrating Vγ9Vδ2 T cell frequency by deconvolution of human cancers microarrays showed that CRC patients with high infiltration of Vγ9Vδ2 T cells had higher overall survival rates (40). These findings indicate that Vδ2 T cells are closely related to the progression and prognosis of tumor patients.

Circulating Vδ2 T cells that are stimulated by microbial phosphoantigens and IL-2 would express gut-homing integrin α4β7 (molecules that mediate Vδ2 T cells trafficking to the human intestinal mucosa) and widely populate the mucosa of the human intestinal tract (41). Moreover, Vδ2 T cells can respond and proliferate rapidly, following stimulation with microbial phosphoantigens, and produce proinflammatory cytokines such as IFN-γ and TNF-α, thus contributing to mucosal immune responses (41). Aminobisphosphonates (42) (zoledronate and pamidronate) induce isopentenyl pyrophosphate (IPP) accumulation in tumor cells, thus activating and amplifying Vγ9Vδ2 T cells (43). Vγ9Vδ2 T cells that were stimulated by zoledronate and exposed to colon cancer stem cells are induced to proliferate and secrete cytokines (TNF-α and IFN-γ), cytotoxic and apoptotic molecules [TNF-related apoptosis-inducing ligand (TRAIL) and BLT esterase], resulting in the capability of killing and lysing colon tumor cells (20). And the cytotoxicity of Vγ9Vδ2 T cells in colon cancer is mainly mediated by the granule exocytosis pathway of effector substances (cytokines and granzymes). Moreover, Maria Raffaella Zocchi et al. (44) found that the local TME of CRC stimulated by zoledronate expresses BTN3A1/CD277 (BTN3A1 can bind to phosphoantigens and drive the activation of Vγ9Vδ2 T cells through conformational changes of the extracellular domains) to stimulate and expand effector Vγ9Vδ2 T cells that carry antitumor activity. Therefore, Vδ2 T cells, especially Vγ9Vδ2 T cells, have the potential to be used in immunotherapy against CRC, and zoledronate serves a stimulator in antitumor immunotherapy to activate and expand γδ T cells.

### Vδ1 T Cells

Compared with Vδ2 T cells, Vδ1 T cells account for a small proportion of γδ T cells. They are mainly distributed on the surface of the mucosa. Although a large number of studies about the antitumor effects of γδ T cells mainly focus on Vγ9Vδ2 T cell subset, it is becoming more evident that Vδ1 T cells also play a critical role in progression of hematological malignancies and some epithelial-derived solid tumors (including CRC) (45–47).

A study showed that cytomegalovirus-induced Vδ1 T cells could inhibit not only the primary colon tumor growth but also the emergence of metastases (48). After that, Wu, Dang et al. (47) found that the freshly isolated human peripheral blood Vδ1 T cells, especially the Vδ1 T cells expanded *ex vivo*, had better cytotoxicity to adherent and sphere-forming colon tumor cells than Vδ2 T cells. The antitumor effect for colon cancer mediated by Vδ1 T cells could be achieved not only by the secretion of cytokines (CD107a, perforin, granzyme B), but also by the direct contact between cells and the cytotoxicity-related receptors and ligands (Fas, death receptor 4/5, MICA/B, and ICAM-1) (47). The findings also showed that adoptive transferred Vδ1 T cells that were expanded by PHA and IL-7 could significantly inhibit tumor growth and prolong survival of colon tumor-bearing mice (47). Also, by comparing the characteristics of γδ T cells in CRC, normal colon tissue, and normal peripheral blood, Meraviglia, S. et al. (49) found that there were 4% of γδ T cells in tumor-infiltrating lymphocytes, and Vδ1 T cells were the dominant γδ T cell subtype in CRC. Comparing to the adjacent normal colon tissues in CRC patients, Vδ1 T cells from tumor tissues produced significantly less IFN-γ, which was likely due to the presence of some identified inhibitory molecules by colon tumor stem cells. In addition, another recent study showed that the human gut-resident intraepithelial Vδ1 T cell subset, that constitutively expressed NKp46, exhibited high antitumor activity against CRC, and the expression of NKp46 on intestinal intraepithelial lymphocytes was associated with high cytolytic potential. The data of this study also showed that lower frequencies of NKp46pos/Vδ1 IELs in tumor-free tissues from CRC patients was associated with a higher risk of metastasis (50). In conclusion, the findings of these studies demonstrate that Vδ1 T cells are involved in the progression of CRC and pave the way for utilizing Vδ1 T cells in anti-CRC immunotherapy.

## Immune Functions of γδ T Cells

### General Functions of γδ T Cells in Cancer

**FIGURE 2:**
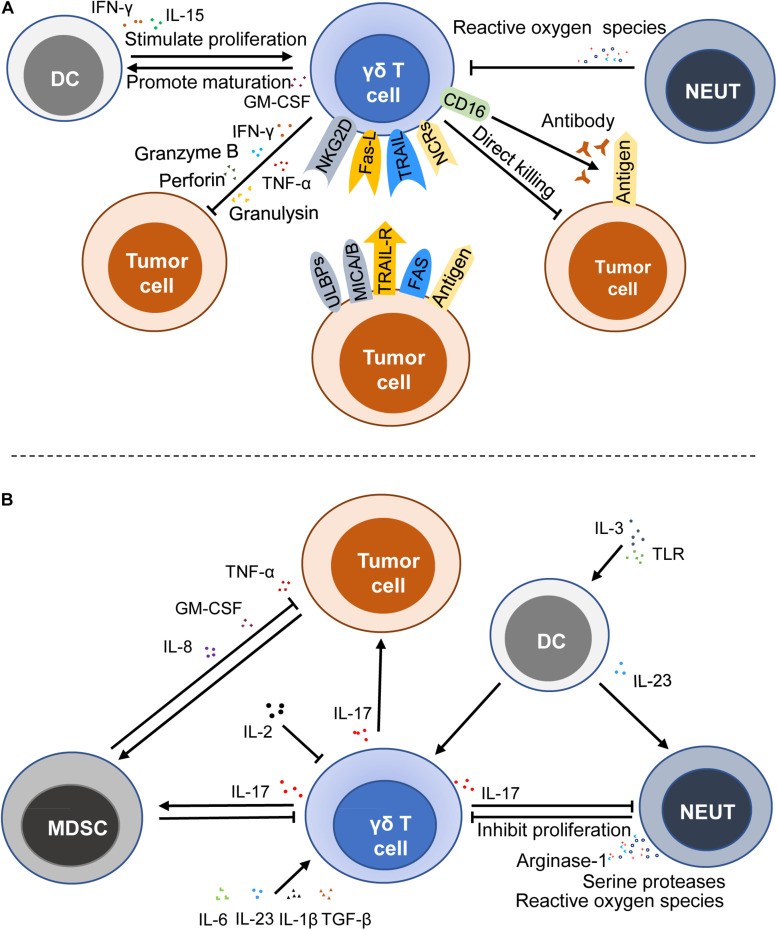
Immune functions of γδ T cells in cancers. γδ T cells can exert an antitumor effect by a direct killing effect, death receptor signal pathway, ADCC, and secreting cytokines and chemokines **(A)**, whereas γδ T cells can stimulate and promote the growth and progression of cancer cells by producing IL-17 **(B)**. MDSC, myeloid-derived suppressor cell; DC, dendritic cell; NEUT, neutrophil; NCRs, NK cell receptors; ULBPs, UL16-binding proteins; NKG2D, natural killer group 2, member D; MICA/B, MHC Class I-related sequence A and B; TRAIL, TNF-related apoptosis-inducing ligand; TRAIL-R, TNF-related apoptosis-inducing ligand-receptor; Fas-L, fas ligand; ADCC, antibody-dependent cytotoxicity; GM-CSF, granulocyte-macrophage colony stimulating factor; TLR, toll-like receptor; TNF-α, tumor necrosis factor-α.

Tumor immunosurveillance is a critical part of immunosurveillance. As a key player in tumor immune surveillance, γδ T cells are able to perceive the changes of the antigens on the malignant cells. The mevalonate biosynthetic pathway is a metabolic pathway of synthetic IPP and dimethylallylpyrophosphate (DMAPP) in general cells. When this pathway in tumor cells was inhibited, it could lead to the accumulation of IPP, thereby activating γδ T cells to play an antitumor role. A recent study focused on CRC has shown that zoledronate, which can suppress the mevalonate biosynthetic pathway, plays a key role in the activation of γδ T cells (44). It is reported that phosphoantigens activate γδ T cells in a TCRs-dependent manner (51), but the concrete mechanism of recognition and activation is still not clear. Furthermore, studies have shown that γδ T cells not only have antitumor effects but also protumor effects, which are mainly mediated by IL-17 (17, 23) (Figure 2).

#### Antitumor Effects and the Mechanism

Previous studies have shown that accumulation of IPP and DMAPP in cancer cells induces abnormal expression of MHC Class I polypeptide-related sequence A and/or B (MICA/MICB) and UL16-binding proteins (ULBPs), thus activating γδ T cells by binding MICA/MICB and ULBPs to NKG2D (a lectin-type activating receptor, which is expressed on the surface of the most NK and NKT cells) (52–54). It is reported that the activation of the cytolytic response of human γδ T cells is associated with the secretion of perforin and granzyme B, which is mediated by NKG2D (55, 56). Besides, Simoes André E. et al. reviewed that γδ T cells also express NK cell receptors (NCRs) like NKp30, NKp44, and NKp46, showing NK cell-like effects to recognize tumor antigens (50, 57). A recent study also demonstrated that the NKp46 expression on γδ intestinal epithelial lymphocytes was associated with high antitumor activity against CRC (50). Since γδ T cells express NCRs and have both T cell characteristics and NK cell characteristics, they are regarded as a bridge between innate immunity and adaptive immunity.

In addition to the recognition and activation mediated by TCRs and NCRs, γδ T cells can be activated by TRAIL. TRAIL receptors often overexpressed and usually located in the endochylema and cell nucleus of tumor cells. Doaa Tawfik et al. (19) found that the knockout of TRAIL-R4 could reduce the sensitivity of cancer cells to cytotoxicity through up-regulation of COX-1 and COX-2 as well as the production of PGE2 that could attenuate the toxic activity and proliferation of γδ T cells. Another death receptor signal pathway is mediated by Fas ligand (FasL) produced by γδ T cells, which induce the killing of target cells *via* the binding with the Fas receptor (22, 58). Antibody-dependent cytotoxicity (ADCC) is also a significant death-inducing mechanism. Vδ2 T cells that express FcγRIIIA (CD16) could kill colon carcinoma through ADCC (51). When tumor antigens are bound to the corresponding antibody, the Fc segment of the antibody will combine with the receptor on the surface of γδ T cells, thereby killing target tumor cells. Moreover, it is reported that IL-15, programmed death receptor-1 (PD-1), and CD19-specific triple body SPM-1 can enhance the ADCC of γδ T cells (59–61). Apart from these, γδ T cells show a direct killing effect on transformed cells by producing perforin, granzymes, and granulysin.

In addition to the above-mentioned cytokines and the corresponding receptors that are able to affect the antitumor activity of γδ T cells, some immunocytes also affect their antitumor immune activity. A recent study has shown that the levels of cytotoxicity-related markers (CD16) and co-stimulatory molecules (CD80 and CD86) are higher when γδ T cells are exposed to IL-15 DCs (16). And IL-15 DCs could stimulate the proliferation of the γδ T cells by inducing the production of soluble IL-15 and IFN-γ and the contact-independent mechanism in the leukemia environment, thus promoting the antitumor activity (16). Activated γδ T cells also promote the maturation of DCs (62). Moreover, the killing capabilities of freshly isolated resting human γδ T cells on ductal pancreatic adenocarcinoma cells was reduced when neutrophils were present, and it was more obvious when neutrophils were activated by zoledronate (63). Although the interaction between these immunocytes and γδ T cells has not been demonstrated in the current CRC studies, many studies have clearly shown that these cells can interact with each other. Therefore, further investigation into the interaction between these cells in CRC is warranted (Figure 2A).

#### Protumor Effects and the Mechanism

IL-17 is an inflammatory cytokine that is mainly produced by activated T cells and can mediate inflammation. In humans, IL-17 is believed to promote angiogenesis and tumor growth by recruiting MDSCs (26, 32, 64) (MDSCs can further promote tumor growth after being attracted and activated by tumor cells). IL-17 producing γδ T cells are recognized as a critical source of IL-17 and play an important role in the tumorigenesis of many cancer types (32–34, 49). IL-17 produced by γδ T cells drives the occurrence and progression of the tumor through several downstream effects on tumor cells, endothelia, and other immunocytes.

Some cytokines, like IL-1β and IL-23, could induce the production of IL-17 by γδ T cells (65). Microbial products are likely to trigger IL-23, which is mainly produced by tumor-associated myeloid cells, to facilitate the tumoral IL-17 response, thereby promoting tumor growth and progression (66). IL-6 and TGF-β promote the production of IL-17 mediated by C5a (26, 67). In addition, the absence of IL-2 can result in higher IL-17 production in IL-2-deficient mice, and IL-2 is able to down-regulate IL-7R to negatively impact the survival of γδ T17 cells (68).

Indeed, the tumor promotion by IL-17 γδ T cells is also regulated by neutrophils, DCs, and other cells. Neutrophils interact with IL-17 γδ T cells and play a critical role in regulating TME. Pioneering work has demonstrated that tumor-infiltrating neutrophils inhibit the proliferation of γδ17 T cells through inducing oxidative stress and restraining the production of IL-17 in TME (69). Hans-Heinrich Oberg et al. (70) noted that neutrophils activated by zoledronate inhibited the γδ T cell proliferation due to not only the production of reactive oxygen species but also the expression of arginase-1 and serine proteases. However, their subsequent study showed that neutrophils co-cultured with γδ T cells could enhance the killing ability of activated IL-17 γδ T cells in the presence of zoledronate, which might be attributed to the fact that direct TCRs-dependent activation enhanced the cytotoxic activity and cytokine/granzyme B production of γδ T cells (63). The direct TCRs-dependent activation is mediated by γδ T cell-specific pyrophosphate antigens or bispecific antibodies. In turn, the activity of neutrophils is influenced by the production of IL-17 and other substances. These studies indicate that the interaction of neutrophils and T cells perhaps depend on the local microenvironment of the tumor. Therefore, further studies are needed to explore the specific factors that can affect the interaction between neutrophils and γδ T cells. In other diseases, it has been proved that IL-23 induced γδ T cells to produce IL-17 and DCs secreting IL-23 could promote the infiltration of neutrophils in stress sites (71). Later, Lo Presti, E. et al. (72) reported that plasmacytoid DCs stimulated by the Toll-like receptor (TLR) and activated by IL-3 could induce the proliferation of Vγ9Vδ2 T cells and selectively induce the production of IL-17 when they were co-cultured with Vγ9Vδ2 T cells. However, there are few reports about the interaction between IL-17 γδ T cells and DCs in CRC, which need to be further investigated in the future (Figure 2B).

### Functions of γδ T Cells in CRC

#### Antitumor Effects

γδ T cells are suggested to inhibit the formation and progression of colorectal adenocarcinoma (73). Tumor-derived γδ T cells that were stimulated by CD3 monoclonal antibodies could produce a lot of IFN-γ and showed strong cytotoxic activities to autologous and allogenic gastrointestinal tumor cells (74). A recent study showed that Phloretin could enhance the cytolytic effects of γδ T cells on colon cancer cells by facilitating the proliferation of IFN-γ producing γδ T cells, and the underlying mechanism might be associated with the increased expression of PFP, GraB, and CD107a as well as the activation of the Wnt signaling pathway (75). Moreover, Vδ2 T cells that expressed FcγRIIIA (CD16) could kill colon carcinoma cells *via* ADCC (51).

A series of *ex vivo* expansion experiments have shown that γδ T cells stimulated by phosphoantigens have robust killing capabilities on CRC (20, 44, 45, 50, 51). Human γδ T cells stimulated by zoledronate produced higher IFN-γ, TNF-α, granzymes, and TRAIL, thus enhancing the killing effect on the colon cancer stem cells, which was mediated by the granule exocytosis pathway and was related to the expression of isoprenoid by tumor cells (20). TCRs expressed on γδ T cells are able to mediate colon cancer stem cell recognition and killing, whereas NKG2D play a role only when tumor targets express several NKG2D ligands (20). Moreover, a recent study suggested that the high cytolytic potential and the production of IFN-γ were relevant to the NKp46 expression on γδ intestinal epithelial lymphocytes, which was associated with the high antitumor activity against CRC (50). Tumor-infiltrating γδ T cells (Vδ 1 and Vδ 2 T cells) expanded *ex vivo* exert strong inhibitory effects and killing activity to rectal cancer cells (17). Furthermore, zoledronate could induce the CRC microenvironment expressing BTN31 to produce effector γδ T cells with anti-CRC activity (44). Altogether, γδ T cells are characterized as effector cells with tumor-killing abilities.

#### Protumor Effects

A recent study has shown that the upregulation of IL-17 is correlated with the colorectal tumorigenesis (66), suggesting that if the expression of IL-17 decreases, the occurrence and development of CRC will be inhibited. Indeed, several studies have confirmed that inhibition of IL-17 suppresses the occurrence of colitis, colonic dysplasia, and colon cancer (64, 76). For example, research by Mahesh Kathania et al. (24) has shown that the ubiquitin ligase Itch can restrain the expression of IL-17 by inhibiting or deactivating ROR-γt ubiquitination, therefore protecting against colitis-associated colon cancer.

In 2014, Pin Wu et al. (32) were the first to report the role of IL-17 γδ T cells in promoting human CRC and demonstrated that γδ T cells are the main source of IL-17 in human CRC. They found that disruption of CRC epithelial barrier resulted in the accumulation and activation of inflammatory DCs, and triggered γδ T17 cells polarization as well as production of IL-17, IL-8, TNF-α, and GM-CSF in γδ T cells, which promoted the recruitment and proliferation of MDSCs, and finally inhibited inflammation triggered by CRC and promoted tumorigenesis (32) (Figure 2). Another research team assessed the secretion of IL-17, IFN-γ, and TNF-α by CRC-infiltrating γδ T cells stimulated by ionomycin and PMA *in vitro*, then used three different FACS gating strategies. Surprisingly, they confirmed that the majority of CD45^+^ IL-17^+^ cells both in CRC and in adjacent non-tumor colon tissues were CD3^+^ cells but not γδ T cells that preferentially produced IFN-γ in CRC and adjacent normal tissues (49). Moreover, the production of IFN-γ by γδ T cells (Vδ 1 and Vδ 2 T cells) was significantly reduced in CRC tissues compared with adjacent normal tissues. The results of this study showed that compared with normal colon tissue and blood, tumor-infiltrating γδ T cells had reduced the capacities to produce IFN-γ but did not produce IL-17 (49). The discrepancy that the results of this study are not consistent with the former study is likely due to some unknown inhibitory components in local TME.

### Functions of γδ T Cells in IBD and CAC

Inflammatory bowel diseases (IBD) including Crohn’s disease (CD) and ulcerative colitis (UC) are complex chronic inflammatory disorders of unknown origin that could affect the intestinal tract (77). Colitis-associated cancer (CAC) may develop in patients with IBD, which is primarily due to chronic intestinal inflammation (78, 79). It is reported that the cumulative incidence of CRC by colitis duration was 2.5% at 20 years, 7.6% at 30 years, and 10.8% at 40 years (78). As the population of patients with IBD grows older, there is an increasing risk of CAC development (80). However, the underlying mechanisms of initiation and development of IBD and how chronic inflammation in IBD leads to CAC development remain unclear. It is well recognized that multiple components of the immune system are involved in the pathogenesis of IBD and CAC (81). γδ T cells, as a critical component of the immune system, are mainly distributed in the mucosa of the intestinal epithelium and regarded as the first line of defense against pathogens, and may play a significant role in the pathogenesis of IBD and CAC (82).

Both human Vδ 1 T cells and Vδ 2 T cells are able to exert an immunoregulatory function and contribute to the pathophysiology of IBD (83–85). Initial studies demonstrated that the number of Vδ 1 T cells were increased in the inflammatory tissue of IBD patients, and Vδ 1 T cells were a major source of IFN-γ (86), suggesting that Vδ 1 T cells played a role in controlling IBD. Moreover, IL-15 produced by epithelial cells plays a key role in γδ T cells, regulating mucosal inflammation in the mouse colon (83). Later, a study showed that the frequency of Vδ 1 T cells in tissue from IBD patients was decreased while Vδ2 T cells were increased in the gut of IBD patients and contributed to TNF-α production (84). Two similar studies also showed that Vδ 2 T cells were the dominant tissue-infiltrating γδ T cells in chronic inflamed IBD, whereas Vδ 1 T cells presented more in healthy colon tissue (85, 87). The tissue-infiltrating Vδ 2 T cells in IBD produced amounts of IFN-γ and TNF-α. Moreover, Lo Presti, E. et al. found that low percentages of Vδ1 and Vδ2 T cells were infiltrated in CAC tissue, but the levels of IFN-γ, TNF-α and IL-17 produced by Vδ2 T cells were increased (85, 87). Transcriptomes also showed a clusterization of gene expression found in IBD patients, which was related to the induction and the maintenance of the inflammatory status (85). The gene expression profile revealed that patients with sustained IBD had an overexpression of the pro-inflammatory cytokine genes (87). These findings demonstrated that Vδ 2 T cells were related to the IBD and CAC pathogenesis and played a protective role in both diseases. The different findings of early studies and later studies, is probably due to the limitation of sample size and the difference of experimental materials as well as methods. Further studies are needed to determine the functions of different γδ T cell subpopulations in the pathophysiology of human IBD. In addition to Vδ 1 T cells and Vδ 2 T cells, Kadivar et al. have defined and characterized a novel subtype of human CD8αβ^+^ γδ T cells that were enriched in the intestinal tract of patients with active IBD and produced cytotoxic mediators, such as IFN-γ and TNF-α, suggesting a role in IBD (88).

## CRC Immunotherapy Based on γδ T Cell

Immunotherapy is another important strategy of antitumor therapy after surgery and adjuvant therapies, which has shown unprecedented success in clinical practice. Application of programmed cell death protein-1/programmed cell death-ligand 1 (PD-1/PD-L1) and chimeric antigen receptor-T (CAR-T) cells, have completely changed the treatment landscape of many different malignancies like leukemia, melanoma and so on (59, 89, 90). However, in solid tumors like CRC, the role of immunotherapy based on PD-1/PD-L1 and CAR-T cells is limited. By contrast, immunotherapy based on innate immunocytes like γδ T cells shows antitumor activity with fewer observed side effects (91, 92). The reaction of γδ T cells to recognize tumor antigens in the MHC-unrestricted manner is rapid, which makes them a key player in immune surveillance. Moreover, γδ T cells could be amplified easily either *in vivo* or *ex vivo.* Studies also reported that the prognosis of cancer patients is related to the percentage of γδ T cells (39, 49). Therefore, it is a feasible antitumor strategy to use γδ T cells against CRC.

### Treatment Approaches

Because tumor phosphoantigens are recognized and activated by TCRs of γδ T cells in an MHC-unrestricted manner (29), phosphoantigens are usually used to stimulate γδ T cells *ex vivo* and *in vivo*. Chemosynthetic phosphoantigens (like bromohydrin pyrophosphate, BrHPP) and aminobisphosphonates (like pamidronate and zoledronate) are able to activate and enhance the cytotoxicity of Vγ9Vδ2 T cells by blocking the mevalonate pathway and promoting the intracellular accumulation of IPP (43). In addition to the stimulation of phosphoantigens, the proliferation of γδ T cells depends on the existence of IL-2. IL-2 is a type of cell growth factor in the immune system and plays an important role in anti-infection and antitumor by: (1) regulating the activity of lymphocytes in peripheral blood; (2) promoting the proliferation of activated T cells; (3) inducing the cytotoxicity of NK cells and cytotoxic T lymphocytes; and (4) enhancing the ADCC against malignant cells *via* stimulating the expression of CD56, IL-2Rα and TNF receptors. Adoptive immunotherapy based on IL-2 has been used in different tumors, such as melanoma, kidney cancer, hepatic carcinoma, and CRC. In addition, early studies have shown that the use of low-dose IL-2 can reduce the recurrence of patients with hematological malignancies treated by bone marrow transplantation (93). Although a high dose of IL-2 may produce a lot of side effects, the adverse reactions caused by a low dose of IL-2 cannot be ignored. For instance, when a low dose of IL-2 stimulates γδ T cells, it will increase the number of circulating regulatory T cells, thus resulting in strong immunologic suppression. Therefore, an accurate dosage and regimen of IL-2 are required to expand γδ T cells when treating patients with tumors.

There are two strategies to activate and amplify γδ T cells. (1) *In vitro* expansion: γδ T cells are isolated from the tissues or peripheral blood of the patients with tumors, stimulated with phosphoantigen, anti-TCRγδ antibody (17) or IL-2, expanded to a certain number, and finally reinfused back to patients to play an antitumor role. This immunotherapy strategy is also known as adoptive transfer, which can expand γδ T cells *ex vivo* to obtain the required number of cells. But this method also has limitations like a high treatment cost and the need for accurate control of the amplification process. (2) *In vivo* expansion: a process by which the circulating γδ T cells are expanded by intravenous administration of phosphoantigens or aminobisphosphonates in the presence of IL-2, is relatively less expensive. However, clinical trials aimed at the *in vivo* expansion of γδ T cells in CRC have not been carried out so far.

### Studies Based on γδ T Cell Immunotherapy

During the past few years, most of the studies about γδ T cells-based immunotherapies mainly focused on Vγ9Vδ2 T cell subtypes, especially on how to activate them *in vivo* or *ex vivo* and how to improve their concentration in peripheral blood. A series of *ex vivo* studies and clinical trials based on γδ T cells against CRC have also been reported in recent years.

Bouet-Toussaint, F. et al. (94) collected peripheral blood samples from 11 tumor patients (six with hepatocellular carcinoma, four with CRC, one with sarcoma) and 16 healthy people. They then amplified Vγ9Vδ2 T cells *ex vivo* with a single dose of phosphoantigen (BrHPP or zoledronate) in the presence of exogenous IL-2. Two weeks later, the expanded Vγ9Vδ2 T cells moderately expressed CD16 (an NK marker related to cytotoxic function) and strongly expressed C-type lectin receptor CD161 (NKRP-1A), NKG2D, and CD94 co-receptor. Moreover, autologous Vγ9Vδ2 T cells stimulated by BrHPP or zoledronate showed specific cytolytic activity against HCC and CRC primary cells but not against autologous primary normal cells, which might be related to the NKG2D expression on Vγ9Vδ2 T cells (94). Moreover, the proliferated Vγ9Vδ2 T cells that were stimulated by BrHPP could selectively recognize and lyse HCC and CRC by releasing IFN-γ and TNF-α. Altogether, it is feasible in adoptive immunotherapy for HCC and CRC, that Vγ9Vδ2 T cells can be expanded from peripheral blood mononuclear cells (PBMCs) of patients with advanced cancer and stimulated by phosphoantigens.

Although Vγ9Vδ2 T cells stimulated by phosphoantigens have lytic activity to a variety of human malignant tumors, Cabillic, F. et al. (43) observed that nearly half of tumor patients showed a low proliferative response of Vγ9Vδ2 T cells after the conventional stimulation of phosphoantigens. Subsequently, they co-cultured PBMCs from HCC patients and CRC patients with hepatic metastases (mCRC) with immature DCs stimulated by aminobisphosphonate zoledronate to amplify γδ T cells *ex vivo*. They found that PBMCs co-cultured with zoledronate-pretreated DCs could induce strong γδ T cell expansion, significantly increased the expression of IPP and IFN-γ, thus enhanced the cytotoxicity of Vγ9Vδ2 T cells. Interestingly, the stimulation of zoledronate-pretreated DCs on Vγ9Vδ2 T cells resulted in higher IFN-γ production than that of BrHPP or zoledronate. The tumor cells pretreated with zoledronate could significantly increase the cytotoxicity of Vγ9Vδ2 T cells against isolated tumor cells from patients. Therefore, Vγ9Vδ2 T cell expansion can be effectively improved through the co-culture of PBMCs with DCs pretreated by zoledronate, indicating that this strategy could be used to improve the efficacy of immunotherapy for HCC and mCRC patients.

The above researches indicated that both phosphoantigens and IL-2 were necessary for the expansion of γδ T cells *ex vivo*. Some clinical studies about Vγ9Vδ2 T cell-based adoptive transfer therapies also showed that IL-2 was necessary for the proliferation of γδ T cells *in vivo* (92). However, a recent study showed that adoptively transferred Vγ9Vδ2 T cells could also be amplified well *in vivo* without the infusion of extrinsic IL-2. The research group of Izumi, T. et al. (42, 95) used zoledronate + IL-2 to prepare a large scale of proliferated Vγ9Vδ2 T cells *ex vivo*. They applied the expanded Vγ9Vδ2 T cells to phase I study of adoptive immunotherapy for patients with recurrent non-small-cell lung cancer, and the patients showed great tolerance to the cultured Vγ9Vδ2 T cells (95). After that, they conducted a clinical trial to evaluate the efficacy of adoptive immunotherapy based on autologous γδ T cells for patients who received pulmonary metastasectomy of CRC. They identified the characteristics of Vγ9Vδ2 T cells in patients who received the adoptive transfer. The number of Vγ9Vδ2 T cells increased gradually in six patients who received autogenous Vγ9Vδ2 T cells injection, and the percentage of IFN-γ^+^ or CD107a^+^ cells in Vγ9Vδ2 T cells was higher than that of other Vγ9-CD3^+^ T cells, suggesting that Vγ9Vδ2 T cells *in vivo* had toxicity against cancer cells. Moreover, in the absence of exogenous IL-2, the *ex vivo*-expanded γδ T cells infused back into the patients showed a CD45RA^–^CD27^–^ effector phenotype and were IL-2Rα-IL-7Rα-IL-15Rα-IL-2Rβ+ γc+ that might bind to the IL-15Rα/IL-15 complex, which suggested that endogenous IL-15 might sustain the proliferation of γδ T cells in patients treated with adoptive transfer (42) (Table 1).

**TABLE 1:** Clinical trials using γδT cells.

**References**	**Year**	**Disease**	**Number of patients**	**Phase of clinical trial**	**Treatment approach**	**Intervention**	**Outcomes**
J. Bennouna et al. (92)	2010	RCC, CC, EC, GC, OC, BC	28	I	Adoptive transfer	BrHPP + IL-2	12 SD, 16 PD
A. Noguchi et al. (91)	2011	BC, CC, LC, others	25	I	Adoptive transfer	Zol + IL-2	3 SD, 8 PD, 3 PR
A. J. Nicol et al. (96)	2011	Melanoma, OC, CC, BC, others	18	I	Adoptive transfer	Zol + IL-2	3 SD, 11 PD, 2 PR, 1 CR, 1 NE
T. Izumi et al. (42)	2013	CRC	6	I	Adoptive transfer	Zol + IL-2	1 CR, 4 PR, 1 NE

*RCC, renal cell carcinoma; CC, colon cancer; EC, esophagus carcinoma; GC, gastric cancer; OC, ovarian cancer; BC, breast cancer; LC, lung cancer; CRC, colorectal cancer; BrHPP, bromohydrin pyrophosphate; Zol, zoledronate; SD, stable disease; PD, progressive disease; PR, partial response; CR, complete response; NE, not evaluable.*

Other several clinical trials based on γδ T cells are also conducted in CRC patients (Table 1). A total of 28 patients with advanced solid tumors, including three colon tumor patients, were enrolled in a two-center, open-label, phase I study, and the results showed that the application of the combination of bromohydrin pyrophosphate (IPH_1101_) and a low-dose of IL-2 was safe and well-tolerated in antitumor immunotherapy, which could effectively induce the expansion of γδ T cells in tumor patients, and the degree of expansion depended on the concentration of IPH_1101_ (92). In this trial, no patient achieved an evaluable objective response; stable disease was observed in 12 patients, and progressive disease was observed in 16 cases. No grade 4 toxicity was observed at any dose level among 28 patients. Similarly, another clinical trial involving 25 patients with advanced solid tumors (including 1 patient with colon cancer) also showed that no severe adverse events were observed after the reinfusion of γδ T cells stimulated and amplified with zoledronate and IL-2 (91). A total of 14 patients were evaluable for objective tumor response. Among these 14 patients, eight cases of progressive disease, three cases of stable disease, and three cases of partial response were observed. In addition, A. J. Nicol et al. (96) enrolled 18 patients with advanced solid tumors (three cases of colon cancer) into a phase I clinical study to evaluate the feasibility and safety of immunotherapy based on *ex vivo* expanded, activated Vγ9Vδ2 T cells. No dose-limiting toxicity was observed, but three cases who received Vγ9Vδ2 T cell reinfusion, while continuing previously ineffective therapy, had disease responses. The clinical outcome of this trial is shown in Table 1.

Altogether, we can see that γδ T cell-based immunotherapy is safe and well-tolerated in tumor patients including CRC patients. γδ T cells-based immunotherapy has so far, however, mainly focused on solid tumors such as melanoma, lung cancer, breast cancer, prostate cancer, and so on, and the results of these trials show great tolerance and safety. The clinical research on solid tumors of hollow organs such as CRC and gastric cancer is still insufficient. Therefore, the application of γδ T cells-based immunotherapies in cavity organ tumors requires substantial research in the future.

## Conclusion Remarks

As a bridge between innate immunity and adaptive immunity, γδ T cells have attracted great interest from the research community because of their rapid recognition of stressed cells in an MHC-unrestricted manner, coupled with their robust killing activity in different tumor types. The killing effects are mainly mediated by direct cytotoxicity, secretion of cytokines, expression of death receptors, and so on. γδ T cells also play a role in perpetuating CRC progression, which is closely related to the survival of CRC patients. Studies have shown that they could significantly inhibit the occurrence and development of CRC. On the other hand, recent studies showed that γδ T cells could promote the occurrence and growth of CRC by secreting IL-17. Their antitumor and protumor effects on CRC perhaps mainly depend on the components of local TME. Compared with αβ T cells, γδ T cells recognize tumor cells *via* an MHC-unrestricted manner and can be expanded *ex vivo* easily, thus being used in tumor immunotherapy. Immunotherapies based on γδ T cells are safe and well-tolerated in patients with solid tumors. However, some patients do not respond to γδ T cells-based immunotherapies. It is therefore necessary to explore the interaction between γδ T cells and tumor cells *in vivo*, and to explore what components affect and regulate this process, as well as exploring approaches to the reasons why some patients did not respond to immunotherapy or showed a low proliferative response of Vγ9Vδ2 T cells after the conventional stimulation of phosphoantigens. Further studies are needed to enhance the immunotherapy strategies on CRC, including better amplification of γδ T cells and a better clinical response. Clinical trials with large sample sizes on *in vivo* expansion and adoptive transfer of γδ T cells in CRC should be carried out to optimize current strategies. Also, the combination of traditional chemotherapy and immunotherapy that may improve patient’s response and overall survival is necessary to be explored to investigate the efficacy and safety. Of note, the interaction and mechanism between γδ T cells and other immunocytes remain unclear, so it will be valuable to elucidate it.

## Author Contributions

RM wrote the manuscript. DY, YG, and RY revised the manuscript. KL directed the project, reviewed, and revised the manuscript. All authors contributed to the article and approved the submitted version.

## Conflict of Interest

The authors declare that the research was conducted in the absence of any commercial or financial relationships that could be construed as a potential conflict of interest.
